# Non-Invasive Wide-Field Imaging of Chip Surface Temperature Distribution Based on Ensemble Diamond Nitrogen-Vacancy Centers

**DOI:** 10.3390/s25061947

**Published:** 2025-03-20

**Authors:** Zhenrong Shi, Ziwen Pan, Qinghua Li, Wei Li

**Affiliations:** 1 College of Mechanical and Vehicle Engineering, Changchun University, Changchun 130022, China; 2 Electronic Information Engineering College, Changchun University, Changchun 130022, China

**Keywords:** nitrogen-vacancy center, temperature imaging, chip surface temperature distribution

## Abstract

With the development of chip technology, the demand for device reliability in various electronic chip industries continues to grow. In recent years, with the advancement of quantum sensors, the solid-state spin (nitrogen-vacancy) NV center temperature measurement system has garnered attention due to its high sensitivity and spatial range. However, NV centers are not only affected by temperature but also by magnetic fields. This article analyzes the impact of magnetic fields on temperature detection. By combining the wide-field imaging platform of optically detected magnetic resonance (ODMR) with a temperature-sensitive structure of thin ensemble diamond overlaid on a quartz substrate, high-sensitivity temperature detection has been achieved. And obtains a sensitivity of approximately 10 mK/Hz^1/2^. By combining a CCD camera imaging system, it realizes a wide field of view of 500 μm^2^, a high spatial resolution of 1.3 μm. Ultimately, this study demonstrates the two-dimensional actual temperature distribution on the chip surface under different currents, achieving wide-field, non-contact, high-speed temperature imaging of the chip surface.

## 1. Introduction

With the development of chip technology, the requirements for device reliability in various electronic chip industries continue to increase. By testing the temperature distribution of chips in practical applications, we can largely assess the degree of chip aging and detect weak areas in advance, which is crucial for evaluating reliability [1,2,3,4,5]. Traditional thermometers, such as thermocouples and infrared thermometers, have low spatial resolution (typically not exceeding 10 µm) and can significantly impact the measured object, limiting their effectiveness in high-spatial-resolution applications [6,7,8]. Fluorescent molecular thermometers, while promising, are greatly affected by illumination intensity, and spectral fluorescence measurement techniques require lengthy integration times, resulting in slow temperature imaging [9]. Therefore, non-contact high spatial resolution and high sensitivity temperature measurement is an increasingly developing research hotspot. In recent years, nitrogen-vacancy (NV) centers in diamond have garnered attention for their high sensitivity and wide temperature range, enabling sub-micrometer temperature measurements [10,11,12], providing a powerful new tool for material research and electronic device applications. Combining NV centers with a wide-field camera for wide-field rapid temperature imaging has garnered widespread attention in the field of temperature measurement.

The rapid development of quantum information science has promoted experimental and theoretical research on temperature sensors based on NV centers in diamond [4,13,14]. This is because diamonds have good biocompatibility, and NV centers exhibit high sensitivity to environmental temperature. Most temperature sensors based on NV centers in diamond rely on optically detected magnetic resonance (ODMR) of NV centers in the presence of a magnetic field [15,16,17], which the temperature can be inferred from the resonance frequency of the ODMR spectrum. NV centers are highly sensitive to temperature due to their zero-field splitting (*D*) value, which shifts linearly with temperature changes. However, the alignment of the bias magnetic field significantly affects the linearity of the optically detected magnetic resonance (ODMR) spectra, which in turn influences temperature-measurement accuracy. At the same time, wide-field imaging is limited by the high thermal conductivity of diamonds [18,19]. Typically, nanodiamond is coated on the surface of the sample to be tested for wide-field imaging, eliminating the influence of thermal conductivity but also damaging the ground state structure of diamond and reducing the fluorescence collection efficiency.

In this study, we innovatively explored two crucial aspects. First, we delved into the relationship between the temperature sensitivity of diamond NV centers and the alignment of the bias magnetic field. A bias field aligned along the <111> axis suppresses off-axis lattice strain, enhancing the linearity of the ODMR spectral shift (Δ*D*). This alignment leads to a 40% increase in sensitivity compared with non-aligned configurations. Secondly, based on this finding, we designed a hybrid diamond quartz device. By integrating a 200 nm NV-doped diamond layer. We successfully achieved wide-field imaging (500 µm^2^) of thermal diffusion on the chip surface, addressing the limitations of existing nanodiamond coating systems.

## 2. Experimental Principles

### 2.1. Principle of Diamond NV Centers Temperature Measurement

**Figure 1:**
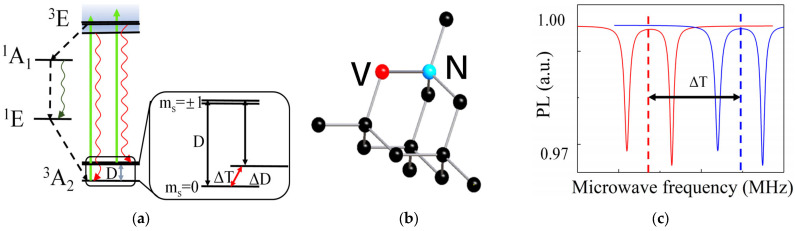
(**a**) Energy-level diagram of the NV centers. (**b**) Diamond NV center crystal structure. (**c**) The ODMR spectral shift caused by temperature.

The NV center in diamond is a point defect characterized by a substitutional nitrogen atom and an adjacent vacant lattice site, exhibiting *C*_3V_ symmetry [20]. This defect has a unique electronic structure that enables high-sensitivity temperature sensing. NV centers can be classified into two types based on their charge states: electrically neutral NV^0^ and negatively charged NV^−^. Due to its excellent stability in photon emission and long spin coherence time [21,22], NV^−^ centers have attracted wide attention in the field of quantum sensing [23,24]. The diamond NV centers that this article focuses on is NV^−^. Unless otherwise specified, all NV centers in the following text refer to NV^−^ centers. The energy levels of the NV centers consist of a ground state (^3^A_2_), an excited state (^3^E), and intermediate states (^1^A, ^1^E), with the ground state exhibiting a zero-field splitting (*D*) of ~2.87 GHz (Figure 1a). The zero-field splitting is highly sensitive to temperature due to lattice expansion and electron–phonon interactions [25]. When a 532 nm laser initializes the NV centers, electrons transition from the ground state to the excited state, emitting red fluorescence upon relaxation. The fluorescence intensity depends on the electron spin state, which can be manipulated using microwaves to achieve spin flipping between the *ms* = 0 and *ms* = ±1 states [26,27]. The ODMR spectrum, obtained by correlating fluorescence intensity with microwave frequency, reveals the zero-field splitting value (*D*), which shifts linearly with temperature (Figure 1c).

The ground state energy level Hamiltonian of the diamond NV center is as follows [28]:

(1) $$\begin{array}{l} H=D\cdot S_{z}^{2}+g\mu_{B}B_{z}S_{z}+E(S_{x}^{2}-S_{x}^{2})+\\ E(S_{x}S_{y}+S_{y}S_{x})+\lambda \cos(\varpi t)S_{x} \end{array}$$

Among them, *D* represents the zero-field splitting value, *E* is the off-axis zero-field splitting value, *S*_x_, *S*_y_, and *S*_z_ represent the Pauli matrices, and the z-axis is parallel to the NV axis. And *μ_B_* represents the Bohr magneton constant:

Usually, a bias magnetic field *B* is used to counteract the off-axis zero-field splitting parameter in diamonds. Since the off-axis zero field parameter *E* of about 100 kHz is much smaller than the zero-field splitting parameter *D* of about 2.87 GHz; therefore, when a bias magnetic field is present, it can be approximated that S_x_ and S_y_ are zero, with the *z*-axis serving as the quantization axis for the NV center. Thus, the Hamiltonian (1) can be represented as:

(2) $$H=D\cdot S_{z}^{2}+g\mu_{B}B_{z}S_{z}+E(S_{x}^{2}-S_{x}^{2})$$

Local stress in diamonds leads to a nonlinear relationship in ODMR temperature detection. Typically, an offset magnetic field needs to be applied to counteract the influence of local stress on diamonds. When the direction of the bias magnetic field aligns with the NV axis, the zero-field splitting parameter *D* value is most affected, which can significantly reduce the *D* value offset caused by lattice expansion and improve detection sensitivity. Therefore, when conducting NV center temperature measurement, it is advantageous for the external magnetic field to be parallel to the NV axis to enhance measurement sensitivity.

The temperature-dependent characteristics of the NV center primarily arise from lattice expansion and electron–phonon interactions. Typically, the correlation between temperature variation Δ*_T_* and the deviation *f_±_* is elucidated using the formula below.

(3) $$f_{\pm }=D+\beta_{T}\Delta_{T}$$

Here, at room temperature, β*_T_* is equal to −74 kHz/K [10], where Δ*T* represents the temperature change. However, the projection of the magnetic field on different NV axes is different; therefore, the frequency shift caused by temperature variation will also be theoretically different. The relative sensitivity is $\frac{\Delta f/f}{\Delta_{T}}=-74\;\mathrm{ppm}/K$, calculated from $\beta_{T}=-74\;\mathrm{KHz}/K$ and f=2.87 GHz.

The formula for temperature sensitivity is as follows [11,29],

(4) $$\eta =\frac{\Delta \omega }{C\sqrt{R}\left|\beta_{T}\right|}$$

Here, *C* represents the contrast of ODMR spectral lines and Δ*ω* represents the line width of ODMR. R represents photon detection efficiency.

### 2.2. Experimental Setup

The diamond NV centers temperature imaging system comprises an optical system, a microwave system, a bias magnetic field system, and a control system. These components work together to achieve high-sensitivity, wide-field temperature imaging of chip surfaces.

The optical system is responsible for initializing the NV centers and collecting fluorescence. A 532 nm laser (MGL-FN-532 laser-1W, Changchun Xingong Optoelectronics Technology Co., Ltd., Changchun, China) is used to excite the NV centers. The laser beam is shaped and directed through an acousto-optic modulator (AOM) and focused onto the diamond sample using a 10× objective lens (*NA* = 0.3). The spatial resolution is calculated as *r* = 1.22*λ*/(2*NA*) = 1.3 μm (*λ* = 670 nm), and the laser excites the diamond below the objective lens to induce fluorescence, where *λ* is the wavelength of the emitted red fluorescence (~670 nm). The fluorescence emitted from the diamond is collected by the same objective lens and directed through a 650 nm high-pass filter to eliminate background light. The filtered fluorescence is then detected by a CCD camera (Basler A602f) with a resolution of 1000 × 1000 pixels and a pixel size of 5 µm × 5 µm. The camera operates at a frame rate of 250 FPS, providing a wide-field imaging area of 500 µm × 500 µm.

The microwave system is used to manipulate the electron spin states of the NV centers. A microwave source generates a signal at ~2.87 GHz, which is amplified to ~30 dBm using a power amplifier. The amplified microwave signal is transmitted to a horn antenna positioned near the diamond sample. The microwave frequency is swept across the ODMR peaks to obtain the fluorescence intensity spectrum, which is used to determine the zero-field splitting value (*D*) and thus the temperature.

The bias magnetic field system is crucial for aligning the magnetic field with the NV axis to enhance measurement sensitivity. The system consists of a three-axis adjustable electromagnet, which provides a uniform and stable magnetic field near the diamond sample. By adjusting the electromagnet, the magnetic field direction can be precisely aligned with the [111] axis of the diamond crystal, minimizing off-axis strain and improving ODMR spectral linearity. The magnetic field strength can be varied from 0 to 500 mT to study its effect on temperature measurement sensitivity.

The control system coordinates the operation of the optical and microwave systems to ensure synchronized data acquisition. An arbitrary waveform generator (AWG) provides TTL pulse signals to control the laser and microwave sources. The AWG triggers the AOM to modulate the laser pulse duration and frequency while also controlling the microwave frequency sweep. The CCD camera is synchronized with the microwave frequency points to capture fluorescence images at specific microwave frequencies. The acquired data are transmitted to a computer for processing and analysis.

**Figure 2:**
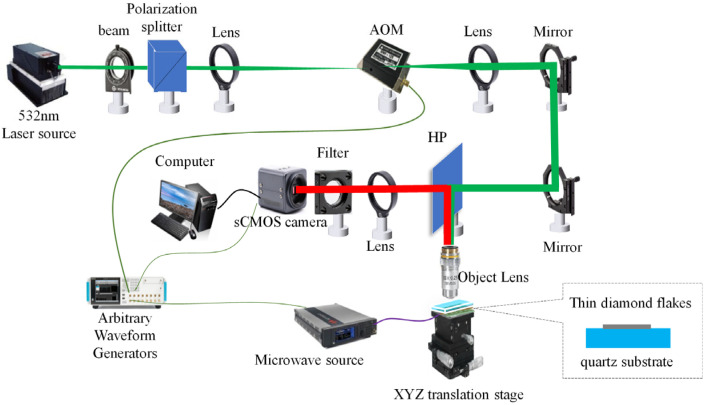
Schematic diagram of experimental setup.

The thermograms were generated using a combination of custom scripts written in Python 3.11 and commercial software (MATLAB, 2022a). The fluorescence intensity data, collected by the CCD camera, was processed to obtain the ODMR spectra. The temperature values were then calculated from the ODMR spectra using the Lorentz fitting formula. The resulting temperature data were used to generate the thermograms, which were further analyzed and visualized using MATLAB, 2022a (see Figure 2).

Diamond NV centers serve as the sensitive unit for temperature detection and are the core of the entire imaging system. Our NV center is in the (110) crystal phase, with a diamond size of 3 mm × 3 mm × 1 mm. Using ion implantation, a high concentration NV centers layer is generated approximately 50 nm below the diamond surface, with a thickness of around 20 um and an NV centers concentration of about 5 ppm. By polishing, we retain a 200 nm thickness of the diamond NV centers, within which the region with the highest NV centers concentration is preserved. We chose quartz as the material for the heat dissipation substrate, as the thermal conductivity of quartz is three orders of magnitude lower than that of diamond, making it both an effective heat dissipation material and not affecting the laser polarization and fluorescent collection of the NV centers. The diamond wafer is bonded to the quartz substrate with crystal bonding wax, serving as the sensitive unit of the imaging system.

## 3. Results

### 3.1. Effect of Bias Magnetic Field Alignment on Temperature Sensitivity

To investigate the impact of bias magnetic field alignment on temperature measurement sensitivity, we conducted experiments with the magnetic field aligned along three crystallographic directions: [111], [110], and [100], as well as a non-aligned configuration (B∥NO). The test object was an aluminum plate controlled by a PID controller, with the CCD collecting 100 × 100 pixels (5 µm^2^ per pixel) over a 50 µm^2^ field of view. The temperature range was set from 300 K to 400 K.

**Figure 3:**
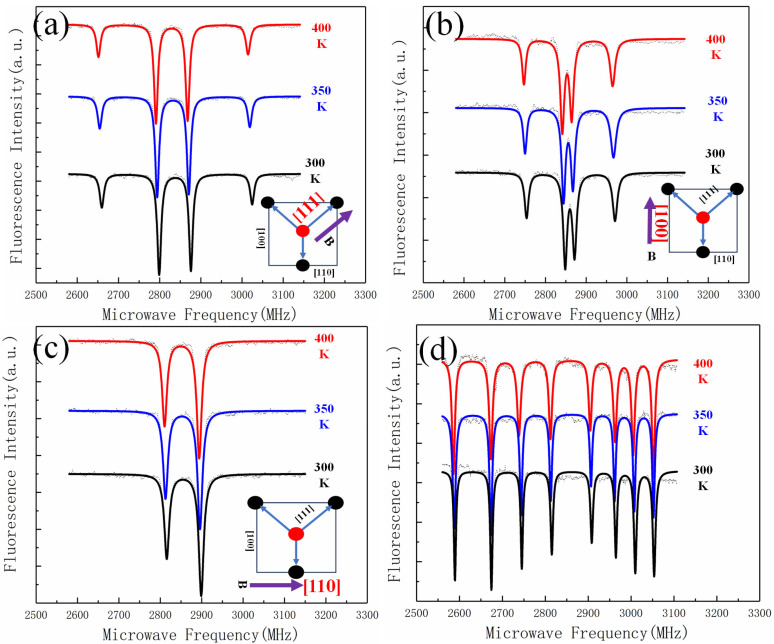
(**a**–**d**) ODMR spectra with *B*∥ <111>, *B*∥<110>, *B*∥<100>, and *B*∥NO at 300 K, 350 K, and 400 K.

The ODMR spectra obtained at 300 K, 350 K, and 400 K for each magnetic field direction are shown in Figure 3. The results indicate that the zero-field splitting value (*D*) shifts with temperature, consistent with the theoretical relationship between temperature and ODMR spectral shifts. However, the magnitude of the *D* value shift varies significantly depending on the magnetic field direction.

The contrast (*C*) and linewidth (Δ*ω*) of the ODMR spectra were measured for each configuration (Table 1). The results show that aligning the bias magnetic field with the [111] axis (*B*∥<111>) yields the highest sensitivity, with a contrast of 12% and a linewidth of 10 MHz. The sensitivity values for each configuration were calculated using Equation (4); the sensitivity values are summarized in Table 1.

**Figure 4:**
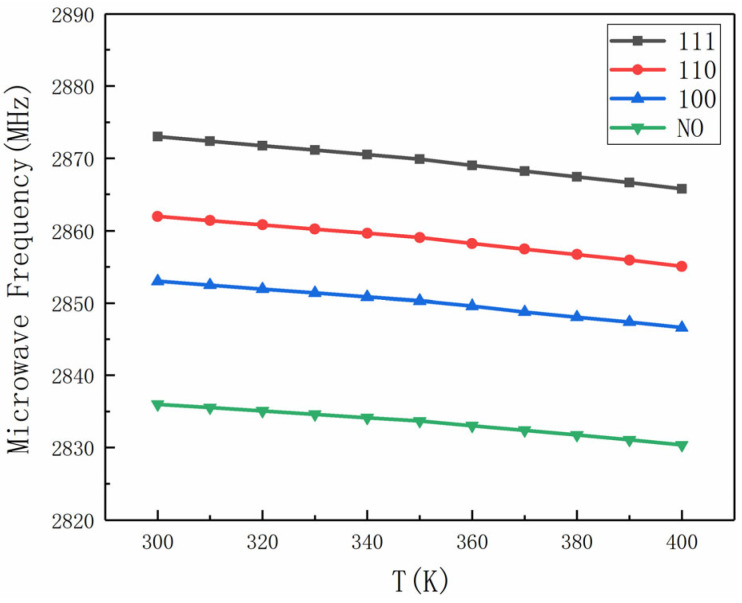
The relationship curve between temperature value T and zero field splitting parameter *D* with *B*∥<111>, *B*∥<110>, *B*∥<100>, and *B*∥NO.

The relationship between the zero-field splitting parameter (*D*) and temperature (*T*) is shown in Figure 4. The slope (β*T*) of the linear fit for each configuration is as follows: *B*∥<111> (−73 kHz/K), *B*∥<110> (−69 kHz/K), *B*∥<100> (−61 kHz/K), and *B*∥NO (−52 kHz/K). These results confirm that aligning the bias magnetic field with the [111] axis significantly enhances temperature measurement sensitivity.

The study investigated the effects of different magnetic field directions on temperature measurement sensitivity. The results showed that the zero-field splitting parameter *D* exhibited a linear relationship with temperature *T*, with varying slopes depending on the magnetic field direction. The sensitivity was highest when the magnetic field was aligned with the [111] axis (*B*∥<111>), achieving a sensitivity of 10 mK/Hz^1/2^.

### 3.2. Wide-Field Temperature Imaging of Chip Surfaces

**Figure 5:**
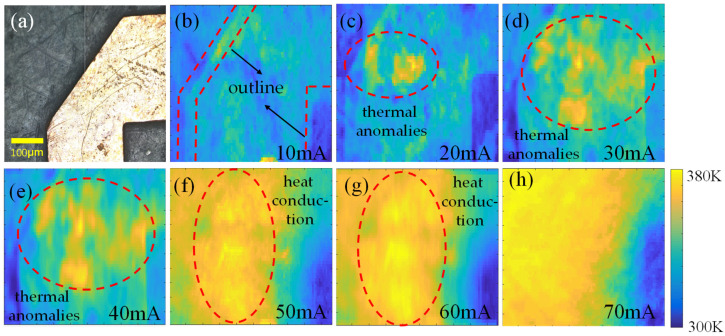
Image of chip surface temperature under different current intensities. (**a**) Physical image of chip surface; (**b**–**h**) the temperature field distribution on the chip surface when the current is 10 mA, 20 mA, 30 mA, 40 mA, 50 mA, 60 mA, and 70 mA, respectively.

To demonstrate the practical potential of the system, we conducted wide-field temperature imaging experiments on a chip based on micro-electromechanical system (MEMS) technology, with the current range from 10 mA to 70 mA. The MEMS chip, fabricated using MEMS technology, is shown in Figure 5a. The CCD camera in the imaging system captured images at a resolution of 1000 × 1000 pixels, with each pixel covering an area of 5 µm^2^, providing a field of view of 500 µm^2^. During the experiment, the bias magnetic field was oriented in the <111> crystal direction, and the imaging system achieved a sensitivity of 10 mK/Hz^1/2^ and a spatial resolution of 1.3 µm. The microwave source was scanned from 2.6 GHz to 2.9 GHz at a step frequency of 0.5 MHz, with the CCD frame rate set at 250 fps and a temporal resolution of 2.4 s.

Under low current conditions (10 mA to 20 mA), the temperature imaging results are shown in Figure 5b,c. The temperature images of the chip clearly revealed its thermal outline. Since the heat generated by the chip was less than the heat dissipation in the environment, the system was able to perform long-term, stable temperature field imaging and identification of the chip. This stable imaging capability provided important support for studying the thermal characteristics of the chip under low-power conditions.

As the current increased further (shown in Figure 5c–e), thermal anomalies began to accumulate in certain regions within the chip. These anomalies were primarily caused by metal defects and surface scratches. Under high current conditions, these defects acted as hotspots, leading to localized temperature increases. At this stage, the system could be used for defect identification and detection in the chip, providing a powerful tool for chip quality control and reliability assessment.

When the current was further increased (as shown in Figure 5f,g), the phenomenon of thermal diffusion within the chip became evident. At this point, the heat accumulation within the chip exceeded the environmental heat dissipation capacity, and heat conduction occurred within the chip. The system was able to perform real-time imaging of the thermal flow under high-temperature conditions. This thermal diffusion phenomenon revealed the complex heat conduction mechanisms within the chip, which is significant for optimizing chip thermal management.

Finally, the thermal imaging test conducted at 70 mA showed a wide temperature gradient of approximately 80 K. At this time, the edges of the metal chip became blurred due to thermal diffusion from the high-temperature regions, consistent with the expected behavior of thermal diffusion in electronic chips.

The experimental results indicate that the system is capable of real-time visualization of the thermal diffusion process and provides valuable insights into the thermal characteristics of chips. In the future, we can optimize the imaging performance in several ways. For example, applying image convolution techniques can effectively reduce the blurring caused by thermal diffusion, thereby enhancing the spatial resolution of the thermal images. Additionally, by optimizing heat dissipation conditions, the system can be tailored to the different operating states of the chips. With its high sensitivity and high spatial resolution, the system is particularly suitable for non-contact, high-speed thermal monitoring in chip development and failure diagnosis.

## 4. Conclusions

This study presents a wide-field temperature imaging system based on ensemble diamond NV centers, achieving high sensitivity (10 mK/Hz^1/2^) and spatial resolution (1.3 µm) over a 500 µm^2^ field of view. The system incorporates two key innovations: (1) aligning the bias magnetic field with the diamond’s [111] axis to suppress off-axis lattice strain, improving ODMR spectral linearity and sensitivity by 40% compared to non-aligned configurations; and (2) a hybrid diamond-quartz device that integrates a 200 nm NV-doped diamond layer with a quartz substrate, mitigating thermal diffusion while preserving fluorescence stability. The experimental results demonstrate the system’s capability to visualize thermal diffusion on chip surfaces under varying currents. The high sensitivity and spatial resolution enable real-time monitoring of temperature distribution, providing valuable insights into chip aging and weak points. These advancements lay the foundation for non-contact, high-speed thermal monitoring in chip development, testing, and fault diagnosis.

## Figures and Tables

**Table 1:** The contrast *C* and line width Δ*ω* of ODMR spectral lines at *B*∥<111>, *B*∥<110>, *B*∥<100>, and *B*∥NO.

	*B*∥<111>	*B*∥<110>	*B*∥<100>	*B*∥NO
Δ*ω* (MHz)	10	11	13	12
*C* (%)	12	12	10	3

## Data Availability

Data are contained within the article.
